# Frequency of Metabolic Syndrome and Study of Anthropometric, Clinical and Biological Characteristics in Peri- and Postmenopausal Women in the City of Ksar El Kebir (Northern Morocco)

**DOI:** 10.3390/ijerph19106109

**Published:** 2022-05-17

**Authors:** Khouloud Harraqui, Dia Eddine Oudghiri, Zineb Hannoun, Hanae Naceiri Mrabti, Sara Aboulghras, Hamza M. Assaggaf, Bodour S. Rajab, Ammar A. Attar, Abdelhakim Bouyahya, Abdellatif Bour

**Affiliations:** 1 Laboratory of Biology and Health, Nutrition, Food and Health Sciences Team, Faculty of Sciences, Ibn Tofail University, Kenitra 14000, Morocco; zineb.hannoun@gmail.com (Z.H.); bour.abdellatif@gmail.com (A.B.); 2 Team UAE/U23FS, Department of Biology, Biology and Health, Faculty of Sciences, Abdelmalek Essaadi University, Tetouan 93002, Morocco; diaeddineoud@gmail.com; 3 Laboratory of Pharmacology and Toxicology, Bio Pharmaceutical and Toxicological Analysis Research Team, Faculty of Medicine and Pharmacy, Mohammed V University in Rabat, Rabat 6203, Morocco; naceiri.mrabti.hanae@gmail.com; 4 Physiology and Physiopathology Team, Department of Biology, Mohammed V University in Rabat, Rabat 10056, Morocco; sara.aboulghras@gmail.com; 5 Department of Laboratory Medicine, Faculty of Applied Medical Sciences, Umm Al-Qura University, Makkah 21955, Saudi Arabia; hmsaggaf@uqu.edu.sa (H.M.A.); bsrajab@uqu.edu.sa (B.S.R.); aaiattar@uqu.edu.sa (A.A.A.); 6 Laboratory of Human Pathologies Biology, Department of Biology, Faculty of Sciences, and Genomic Center of Human Pathologies, Mohammed V University in Rabat, Rabat 10106, Morocco

**Keywords:** dyslipidemia, body mass index, peri menopause, post menopause, diabetes, abdominal obesity

## Abstract

This study aimed to determine the frequency of metabolic syndrome and to identify its predictive factors in peri- and post-menopausal women in the city of Ksar El Kebir, in northern Morocco. A total of 373 peri- and post-menopausal women between 45 and 64 years old participated in the study. Metabolic syndrome was diagnosed according to the National Cholesterol Education Program Adult Treatment Panel III (NCEP-ATP III) definition. Body mass index (BMI) was calculated to assess the degree of obesity in women; anthropometric, clinical and biological parameters were collected during interviews. The mean ages of peri- and postmenopausal women were 48.84 ± 2.4 years and 56.65 ± 4.29 years, respectively. Postmenopausal women had higher means of anthropometric and biological parameters than peri-menopausal women. We also noted a predominance of metabolic syndrome in postmenopausal women (*n* = 158) compared to peri-menopausal women (*n* = 81). Waist circumference was the predominant marker in the subjects studied, whereas triglycerides were the lower marker. In the overall population, the incidence of metabolic syndrome and its associated factors were higher in postmenopausal women than in peri-menopausal women, from which it can be concluded that post menopause may be a predictor of metabolic syndrome.

## 1. Introduction

Morocco is an economically emerging country, and this economic transition through which it is going, in association with other widely documented transitions, such as nutritional, food and demographic transitions, result in an epidemiological transition. The latter is manifested by the appearance of non-communicable chronic pathologies, which go unnoticed and have settled in the Moroccan population and accumulated silently, to such an extent that this Moroccan population is the focal point of our research—it translates this ignorance through specialists and practitioners, of this set of pathologies, formerly known as syndrome X, then insulin resistance, and finally, metabolic syndrome (passing, of course, through changes in other concepts).

Importantly, the relevant point of our research is that the Moroccan population is exposed to this syndrome, including children. To the best of our knowledge, there is no real scientific research with a high degree of scientific evidence, except for occasional research in the context of thesis reports, end-of-university study projects and scientific and administrative reports.

Metabolic syndrome is defined as a group of morphological, physiological, and biochemical disorders, which can increase the risk of type 2 diabetes by ninefold [1,2,3,4], increase the risk of cardiovascular events by threefold [5,6,7], and it can also increase mortality rates [8]. Metabolic syndrome results in abdominal obesity, insulin resistance, hypertension, dyslipidemia, prothrombotic, and proinflammatory states [9]. Its prevalence varies according to the definition used, the year of the study, the characteristics of the population considered, as well as the ethnic groups [10].

Menopause is a normal physiological phenomenon that occurs in all women and encompasses the peri-menopausal and post-menopausal periods. The climacteric or critical age of menopause is between 45 and 65 years of age [1]. It is characterized by the permanent cessation of menstruation due to the loss of ovarian follicular function [2], and it is marked by biological, social and psychological changes [3]. Peri-menopause is considered as a period of cycle irregularity and changes in ovarian activity that precedes the last menstrual period and continues for one year afterwards until post menopause [2,4]. Post menopause is the period beginning 12 months after the definitive cessation of menses [5].

Moreover, the transition from peri to post menopause is associated with the emergence of many characteristics of metabolic syndrome (abdominal obesity, reduction in HDL-C, fasting hyperglycemia, etc.), which explain the progressive increase in its incidence during the period of menopause [11,12].

However, changes in behavior, lifestyle, diet and social environment are associated with an increased prevalence of metabolic syndrome, making it a major public health problem [13]; as a result, the prevention and management of this syndrome are mainly based on the application of lifestyle and dietary measures to reduce obesity or maintain a normal weight and on regular physical activity [7].

The objective of the present work is to determine the frequency of metabolic syndrome and to study its association with the anthropometric, clinical and biological characteristics in a population of peri- and post-menopausal women from the city of Ksar El Kebir in northern Morocco (a country in nutritional, demographic and epidemiological transition).

## 2. Materials and Methods

### 2.1. Study Setting and Participants

Our study is an epidemiological study that took place in the department of Medicine at the hospital of Ksar el Kebir, located in northern Morocco.

The survey was conducted between January 2019 and February 2020, on a sample of 373 women aged 45 to 64 years and divided into two groups: peri-menopausal women and post-menopausal women. Before starting the survey, authorization from the regional delegate of the Ministry of Health and the director of the hospital of Ksar el Kebir was obtained.

The women who visited the hospital were given a clear and detailed explanation of the framework and purpose of the study as well as the steps they would follow, and those who voluntarily signed the consent form were selected to participate in the study. Anonymity and confidentiality were respected.

Inclusion criteria

− Peri-menopausal and post-menopausal women;
− Aged between 45 and 64 years;
− Be of Moroccan nationality and resident in Ksar el Kebir;
− Signed informed consent from the participants.

Exclusion criteria

− Pregnant or breastfeeding women;
− Women who are not residents of Ksar El Kebir;
− Women who did not sign the consent form;
− Women on medication that may affect the results;
− Women suffering from serious chronic diseases (cancer, renal failure…).

### 2.2. Variables

The variables studied for the investigation were: age, weight, height, body mass index (BMI), waist circumference (WC), hip circumference (HC), WC/HC ratio, blood pressure, fasting blood glucose and serum lipid levels (total cholesterol, HDL cholesterol, LDL cholesterol and triglycerides).

### 2.3. Data Collected

#### 2.3.1. Blood Pressure

Blood pressure (BP) was measured with an electronic sphygmomanometer. BP measurements for each participant were taken in the sitting position, after 15 min of rest on both arms, and on three occasions at 5 min intervals. The value retained was the average of the last two measurements. The measurements and the interview took place in a room reserved for the participants. Their confidentiality and privacy were respected. For the illiterate participants, their answers were recorded by the authors according to the participants’ choices.

#### 2.3.2. Anthropometric Parameters

Weight (in Kg) was measured using a Seca mechanical scale.

Height (in m) was determined using a Seca mobile stadiometer. The body mass index (BMI) in (Kg/m^2^) was calculated by dividing the weight (in Kg) by the square of the height (in m). The waist circumference (WC) (in cm) and hip circumference (HC) (in cm) were measured with a Seca brand tape measure directly on the skin in a fully erect position. The WC (in cm) was measured at the midpoint between the lower costal margin and the anterior superior iliac spine on the midaxillary line, and the HC (in cm) at the widest circumference on the buttocks. The WC/HC ratio was calculated by dividing the waist circumference in (cm) by the hip circumference in (cm). The percentage of body fat was measured by the impedance meter [14]. Participants stood barefoot and in light clothing.

The measuring instruments were checked daily.

#### 2.3.3. Biochemical/Biological Parameters

Blood samples were taken by superficial venipuncture at the elbow. The participants came fasting (12 h of fasting). The blood samples thus obtained were used after centrifugation (10 min) on the same day for the determination of fasting blood glucose and lipid parameters: triglycerides, total cholesterol, HDL cholesterol (HDL-C) and LDL cholesterol (LDL-C). The blood samples were taken by nurses from 9:00 to 10:30 in the morning, and the measurements were taken by the laboratory technicians at the hospital of Ksar El Kebir.

### 2.4. Evaluation Criteria

#### 2.4.1. Assessment of Menopause

Participants with irregular menstrual cycles were considered peri menopausal. Participants with a cessation of menses for at least 12 months were considered postmenopausal.

The age at the last menstrual period was recorded.

#### 2.4.2. Assessment of Obesity

Overweight and underweight were defined using body mass index (BMI in kg/m^2^) according to the World Health Organization (WHO) classification [15]: BMI < 18.5 = underweight; 18.50 < BMI < 24.99 = normal weight; 25 < BMI < 29.99 = overweight; 30 < BMI < 34.99 = obese (class I); 35 < BMI < 39.99 = massive obesity (class II); BMI > 40 = morbidly obese (class III).

#### 2.4.3. Diagnosis of Metabolic Syndrome

Metabolic syndrome was diagnosed according to the National Cholesterol Education Program Adult Treatment Panel III (NCEP-ATP III) definition criteria [16] by the presence of at least three out of five of the following risk factors:

- Waist circumference > 102 cm for men and >88 cm for women.
- Triglycerides (TG) ≥ 150 mg/dL.
- HDL cholesterol (HDL-C) < 40 mg/dL in men and <0.50 mg/dL in women.
- Systolic blood pressure ≥ 130 mmHg, Diastolic blood pressure ≥ 85 mmHg.
- Fasting blood glucose ≥ 1.10 mg/dL.

### 2.5. Data Analysis

The collected data were entered and coded in Excel. Then, statistical analysis was performed using SPSS (Statistical Package for Social Sciences) version 26.0. Quantitative variables were presented as mean and standard deviation, while qualitative variables were presented as numbers and percentages. The non-parametric Mann–Whitney test was used to compare continuous variables, and the Chi-square test was used for categorical variables. Correlations between variables were calculated by the Spearman test.

A *p*-value of less than 0.05 was considered the threshold for statistical significance.

The study was conducted in accordance with the criteria of the Declaration of Helsinki (World Medical Association, 2008) [17].

## 3. Results

Of a total of 373 women recruited in the menopausal period, 160 were peri-menopausal women and 213 were postmenopausal women. The mean age (±standard deviation) of the peri-menopausal population was 48.84 ± 2.4 years, and that of the post-menopausal population was 56.65 ± 4.29 years.

The postmenopausal women had almost the same means of anthropometric parameters, namely waist circumference, hip circumference, weight, BMI and body fat, and biological parameters, namely fasting blood glucose, triglycerides, total cholesterol, LDL-C and TG/HDL, as the peri-menopausal women. In contrast, the mean HDL-C remained close between the two groups (Table 1).

In peri-menopausal women, the presence of metabolic syndrome (50.62%) was compared to the absence of metabolic syndrome (49.38%). Indeed, there was a predominance of metabolic syndrome (MS) in postmenopausal women (74.18%) compared to peri-menopausal women.

Anthropometric, lipid and hemodynamic parameters had the highest mean levels in the study population with metabolic syndrome (Table 2).

Waist circumference was the predominant marker in the subjects studied, while triglyceride was the lower marker.

An increase in HDL cholesterol was noted in the postmenopausal group, followed by an increase in hypertension and fasting blood glucose. The HDL cholesterol marker was also found to be elevated in the peri-menopausal group, although their hypertension and fasting blood glucose were low (Table 3).

In peri-menopausal women, fasting blood glucose, SBP and TG/HDL-C showed better positive and significant correlations with metabolic syndrome. Total cholesterol, triglyceride and BMI showed moderate and significant correlations. The weak correlations were found between the syndrome metabolic rate and age, waist circumference, hip circumference, body fat, DBT and LDL-C.

In post-menopausal women, TG/HDL-C, DBT, fasting blood glucose, total cholesterol and triglyceride showed better positive and significant correlations with metabolic syndrome. Moderate and significant correlations were found between metabolic syndrome and waist circumference, BMI and LDL-C. Body fat, hip circumference, and DBT showed weak significant correlations.

The only negative correlations were observed with HDL-C and height in the entire study population (Table 4).

## 4. Discussion

This study assessed the frequency of metabolic syndrome using the NCEP-ATP III definition and investigated its association with anthropometric, clinical and biological characteristics in a Moroccan population composed of peri-and post-menopausal women, aged between 45 and 64 years old and living in Ksar El Kebir. It showed that the frequency of metabolic syndrome differs according to the menopausal status of the participants, being higher in post-menopausal women. Moreover, the increase in body mass index, the fasting blood glucose, the waist circumference, the hip circumference, the blood pressure, the body fat and the serum lipid levels, except HDL-C, were significantly associated with the presence of metabolic syndrome. The evaluation of metabolic syndrome markers indicated that waist circumference and HDL-C were the predominant components in the study population.

The mean age of the peri-menopausal women in this study was 48.84 ± 2.4 years, lower than the mean age (52.6 ± 4.4 years) reported by Flor-Alemany et al. [18] and the mean age of post-menopausal women was 56.65 ± 4.29 years, close to the mean age (57.11 ± 6.07 years) reported by a study on the prevalence of metabolic syndrome in peri- and post-menopausal women attending a tertiary clinic realized in Turkey (57.11 ± 6.07 years) [9]. Our results indicated that the mean BMI of postmenopausal women (31.91 ± 5.44 kg/m^2^) is higher than that of peri-menopausal women (29.93 ± 4.58 kg/m^2^), and weight and BMI increase with peri- and post-menopausal status. The studies of Wing et al. [19] and Macdonald et al. [20] agree with our results, where weight gain is related to peri- and post-menopausal status, in contrast to Pasquali et al. [21], who found that weight and BMI are lower in the post-menopausal period [21]. The mean values of total cholesterol, triglycerides and LDL-C were higher in post-menopausal women than in peri-menopausal women and this difference was statistically highly significant (*p* < 0.001); similar results have been reported by other authors, including de Aloysio et al. in Italy [5], Stevenson et al., in England [22], and Gomina et al. in Benin [23]. The increase in the levels of these lipid parameters during the transition from peri-menopausal to post-menopausal status was also observed in a study on the effect of menopause on lipid profile in relation to body mass index [24]. This transition may cause adverse effects on the serum lipid concentrations of peri- and post-menopausal women [21]. Derby et al. [25] agreed with our results, showing that the lipid profile increases with increasing age and BMI. However, the results from Pasquali et al. [26] and Matthews et al. [27] are in contrast to our results. HDL-C increased during the postmenopausal period, and this result is consistent with those in the literature [25,28,29], in contrast to other studies that showed a reduction in HDL-C [30,31].

Body fat was higher in postmenopausal women, with studies by Rico et al. [32] and Svendsen et al. [33] finding similar results; in contrast, other studies found no difference in body fat between peri- and postmenopausal women [34,35,36].

In our study, according to the NCEP-ATP III definition, the frequency of post-menopausal women with metabolic syndrome (74.18%) was higher than that of peri-menopausal women (50.62%). This result is consistent with other results reported in studies from Thailand [37], Tunisia [38], and Brazil [39]. The study of Ebrahimpour et al. [40] revealed that the frequency of metabolic syndrome in women differs according to their menopausal status; it is significantly higher in post-menopausal women [40], which is also consistent with our results. In addition, it was clearly observed that the incidence of metabolic syndrome increases significantly with age during post menopause (*p* < 0.005). Moreover, the same observation was revealed by Marchi et al. [39], which indicates a statistically significant relationship between increasing age and post-menopausal status with the presence of metabolic syndrome [39]. This is close to the result of other authors who considered age to be a main risk factor for the increase in metabolic syndrome [41].

In post-menopausal women with metabolic syndrome, body mass index, fasting blood glucose, waist circumference, hip circumference, blood pressure, total cholesterol and triglyceride levels were found to be significantly higher than in post-menopausal women without metabolic syndrome (*p* < 0.001), for all the parameters mentioned above, and our results agree with those obtained by Toprakci Sahin et al. [42], and Marjani et al. [43]. The same results were found in peri-menopausal women in our study and in another study conducted in Iran [44].

Both groups of peri- and post-menopausal women with metabolic syndrome had significantly higher LDL-C levels than those without metabolic syndrome (*p* < 0.001), which is comparable with the results shown by two authors cited above [43,44]. Toprakci Sahin et al. [42], in contrast, found a higher LDL-C level in the post-menopausal group and without metabolic syndrome. A decrease in HDL-C level was observed in both peri- and post-menopausal women and with metabolic syndrome, which corroborates with the results of several studies [42,43,44,45]. HDL-C levels in our population were higher in postmenopausal women compared with peri-menopausal women with metabolic syndrome; this result was also found in other studies [46,47].

In their study, Mesch et al. [48] reported that the triglyceride/HDL-cholesterol ratio was considered a surrogate marker of insulin resistance, which is easy to determine and can be used to assess patients at risk for metabolic syndrome. McLaughlin et al. [49] agrees with them on this ratio, which has been among the most useful metabolic markers for identifying insulin-resistant individuals, with a cut-off value above 1.8 used for identification. Insulin resistance is described as a factor involved in the pathophysiology of metabolic syndrome [39], involved in the increase in glucose intolerance and diabetes, hypertension, triglyceride levels and in the reduction in HDL-C levels [50].

In our population, the triglyceride/HDL-cholesterol ratio was significantly elevated (>1.8) in peri- and post-menopausal women with metabolic syndrome (*p* < 0.001), and also elevated in peri- and post-menopausal women without metabolic syndrome (*p* < 0.001). In comparison with our results, the study by Mesch et al. [48] found an elevated triglyceride/HDL-cholesterol ratio in peri- and post-menopausal women but without reaching the threshold value at which women will show insulin resistance.

The literature suggests a change in hormonal secretion and loss of estrogen with menopause, which causes an accumulation of visceral fat, leading to abdominal obesity [51]; other investigators have also shown a change in fat distribution and increase in abdominal adiposity during menopause [52,53]. Abdominal obesity and/or high waist-to-hip ratio are largely responsible for insulin resistance and the development of metabolic syndrome [54], and are positively associated with an increased likelihood of metabolic syndrome [55]. Our results agree with this trend; indeed, abdominal obesity is significantly elevated in our healthy population with metabolic syndrome (*p* < 0.002), but there was a clear increase in waist circumference, especially in post-menopausal women compared to peri-menopausal women with metabolic syndrome. Several other studies also obtained similar findings, where abdominal obesity was high in post-menopausal women with metabolic syndrome [43,56,57].

Post-menopausal women had the significantly highest body fat and hip circumference compared to peri-menopausal women with metabolic syndrome (*p* < 0.003). Joo et al. [57], in their study, made the same conclusions. In our study, abdominal obesity and HDL-cholesterol were the most frequent markers of metabolic syndrome identified in the whole study population, followed by hypertension and fasting blood glucose. The results obtained in these studies corroborate ours [39,57,58]. The correlation between metabolic syndrome and various biological parameters in peri- and post-menopausal women showed that triglyceride/HDL-C ratio, systolic blood pressure and fasting blood glucose showed better and statistically significant (*p* < 0.001) positive correlation; total cholesterol and triglyceride showed better correlation in post-menopausal women, but rather moderate correlation in peri-menopausal women. Waist circumference and BMI also showed a moderate correlation in post-menopausal women. Weak correlations were observed with body fat, hip circumference and diastolic blood pressure. In contrast, HDL-C and height were the only parameters to show a negative correlation. The study by Mesch et al. [48] found that the triglyceride/HDL-C ratio and waist circumference showed the best correlation with metabolic syndrome.

Despite its remarkable findings, this study has some limitations that should be noted. The first limitation includes the cross-sectional nature of the design that does not elucidate causal inferences. The second limitation is the homogeneous sample with an insufficient size, which limits the generalization of the results obtained for all Moroccan postmenopausal women or for other ethnically and culturally different groups of women. Longitudinal studies should be conducted with a larger and heterogeneous sample to validate the results and provide more information. Third, the menopausal status of the study population was unclear due to the lack of serum hormone measurements and assessment. Further, another limitation in this study was the exclusion of menopausal women due to surgery, medical treatment or other causes.

## 5. Conclusions

Our study highlighted the importance of assessing the health status of peri- and post-menopausal women. The conclusions concerning the association between anthropometric, biological and clinical characteristics and metabolic syndrome in these populations are an increase in fat mass, with a significantly elevated presence of waist circumference; also, biological parameters, as well as metabolic syndrome, are higher in post-menopausal women than in peri-menopausal women, and this finding could very likely lead to more severe consequences if hygienic measures are not taken for this category of women.

## Figures and Tables

**Table 1:** Biological characteristics in peri- and post-menopausal women.

	Peri-Menopause (*n* = 160)	Post-Menopause (*n* = 213)	*p*-Value
Age (years)	48.84 ± 2.4	56.65 ± 4.29	0.000 *
Height (cm)	158.79 ± 6.25	157.95 ± 6.07	0.166
Waist size (cm)	99.12 ± 10.45	104.6 ± 11.5	0.000 *
Hip size (cm)	106.17 ± 9.86	109.66 ± 11.39	0.003 *
Weight (kg)	75.94 ± 12.51	79.71 ± 15.21	0.020 *
BMI (kg/m^2^)	29.93 ± 4.58	31.91 ± 5.44	0.000 *
Body fat (%)	39.65 ± 5.88	41.51 ± 5.91	0.001 *
Fasting blood glucose (g/L)	1.2 ± 0.49	1.34 ± 0.53	0.000 *
Total Cholesterol (g/L)	1.7 ± 0.55	1.97 ± 0.68	0.000 *
Triglycerides (g/L)	1.04 ± 0.51	1.35 ± 0.68	0.000 *
HDL-C (g/L)	0.42 ± 0.07	0.43 ± 0.06	0.269
LDL-C (g/L)	1.03 ± 0.45	1.24 ± 0.54	0.000 *
TG/HDL-C	2.54 ± 1.39	3.23 ± 1.68	0.000 *
CRF (bpm)	72.83 ± 9.13	74.77 ± 9.82	0.020 *
SBP (mmHg)	127.56 ± 16.33	132.13 ± 16.2	0.001 *
DBP (mmHg)	76.41 ± 11.86	78.64 ± 11.54	0.039 *

BMI = Body Mass Index; HDL-C = HDL-Cholesterol; LDL-C = LDL-Cholesterol; TG/HDL-C = Triglycerides to HDL-Cholesterol ratio; CRF = Resting Heart Rate; DBP = Dystolic Blood Pressure. Values are presented as means ± standard deviations. * Significant: *p* < 0.05.

**Table 2:** Biological characteristics in peri- and post-menopausal women grouped according to metabolic syndrome (NCEP criteria).

	Peri-Menopause		
	Absence of the MS (49.38%)	Presence of the MS (50.62%)	*p*-Value
Age (years)	48.71 ± 2.56	48.96 ± 2.24	0.245
Height (cm)	159.27 ± 6.33	158.33 ± 6.17	0.453
Waist size (cm)	96.16 ± 9.51	102 ± 10.57	0.001 *
Hip size (cm)	102.89 ± 9.19	109.37 ± 9.47	0.000 *
Weight (kg)	71.94 ± 9.86	79.84 ± 13.6	0.000 *
BMI (kg/m^2^)	28.18 ± 3.09	31.63 ± 5.14	0.000 *
Body fat (%)	38.16 ± 5.75	41.11 ± 5.67	0.002 *
Fasting blood glucose (g/L)	0.95 ± 0.32	1.43 ± 0.51	0.000 *
Total Cholesterol (g/L)	1.47 ± 0.29	1.92 ± 0.64	0.000 *
Triglycerides (g/L)	0.82 ± 0.24	1.25 ± 0.6	0.000 *
HDL-C (g/L)	0.45 ± 0.08	0.4 ± 0.05	0.000 *
LDL-C (g/L)	0.86 ± 0.27	1.21 ± 0.52	0.000 *
TG/HDL-C	1.86 ± 0.6	3.19 ± 1.61	0.000 *
CRF (bpm)	68.99 ± 5.66	76.58 ± 10.28	0.000 *
SBP (mmHg)	118.96 ± 11.59	135.95 ± 15.96	0.000 *
DBP (mmHg))	72.41 ± 9.39	80.31 ± 12.74	0.000 *
	**Post-Menopause**		
	**Absence of the MS (25.82%)**	**Presence of the MS (74.18%)**	***p*-Value**
Age (years)	55.2 ± 4.54	57.16 ± 4.09	0.004 *
Height (cm)	158.25 ± 5.61	157.85 ± 6.23	0.628
Waist size (cm)	96.09 ± 7.72	107.56 ± 11.13	0.000 *
Hip size (cm)	102.84 ± 7.77	112.03 ± 11.5	0.000 *
Weight (kg)	71.31 ± 10.91	82.63 ± 15.44	0.000 *
BMI (kg/m^2^)	28.22 ± 3.52	33.2 ± 5.41	0.000 *
Body fat (%)	37.81 ± 5.64	42.8 ± 5.46	0.000 *
Fasting blood glucose (g/L)	0.98 ± 0.3	1.46 ± 0.54	0.000 *
Total Cholesterol (g/L)	1.44 ± 0.28	2.16 ± 0.68	0.000 *
Triglycerides (g/L)	0.76 ± 0.23	1.55 ± 0.67	0.000 *
HDL-C (g/L)	0.45 ± 0.07	0.42 ± 0.05	0.002 *
LDL-C (g/L)	0.84 ± 0.24	1.38 ± 0.55	0.000 *
TG/HDL-C	1.72 ± 0.5	3.75 ± 1.63	0.000 *
CRF (bpm)	69.35 ± 6.57	76.66 ± 10.08	0.000 *
SBP (mmHg)	117.11 ± 11.32	137.36 ± 14.28	0.000 *
DBP (mmHg))	71.15 ± 8.67	81.25 ± 11.29	0.000 *

BMI = Body Mass Index; HDL-C = HDL-Cholesterol; LDL-C = LDL-Cholesterol; TG/HDL-C = Triglycerides to HDL-Cholesterol ratio; CRF = Resting Heart Rate; SBP = Systolic Blood Pressure; DBP = Diastolic Blood Pressure; MS: metabolic syndrome. Values are presented as means ± standard deviations. * Significant: *p* < 0.05.

**Table 3:** Distribution of metabolic syndrome markers in peri- and post-menopausal women according to NCEP criteria.

	Perimenopause	Postmenopause	*p*-Value
Waist size (>88 cm)	142 (38)	200 (54)	0.075
Triglyceride (≥150 mg/dL)	22 (6)	79 (21)	0.000 *
HDL-Cholesterol (<50 mg/dL)	119 (32)	183 (49)	0.005 *
Hypertension (≥130/85 mmHg)	69 (18)	131 (35)	0.000 *
Fasting blood glucose (≥110 mg/dL)	64 (17)	125 (34)	0.000 *

Values are presented as numbers and percentages in parentheses. * Significant: *p* < 0.05.

**Table 4:** Correlation between biological characteristics and metabolic syndrome in peri- and postmenopausal women.

	Metabolic Syndrome			
	Peri-Menopause		Post-Menopause	
	r-Value	*p*-Value	r-Value	*p*-Value
Age (years)	0.092	0.246	0.198	0.004 *
Height (cm)	−0.060	0.455	−0.033	0.629
Waist size (cm)	0.272	0.001 *	0.464	0.000 *
Hip size (cm)	0.316	0.000 *	0.371	0.000 *
Weight (kg)	0.302	0.000 *	0.331	0.000 *
BMI (kg/m^2^)	0.361	0.000 *	0.427	0.000 *
Body fat (%)	0.247	0.002 *	0.384	0.000 *
Fasting blood glucose (g/L)	0.576	0.000 *	0.526	0.000 *
Total Cholesterol (g/L)	0.406	0.000 *	0.535	0.000 *
Triglycerides (g/L)	0.406	0.000 *	0.540	0.000 *
HDL-C (g/L)	−0.297	0.000 *	−0.216	0.002 *
LDL-C (g/L)	0.384	0.000 *	0.476	0.000 *
TG/HDL-C	0.499	0.000 *	0.585	0.000 *
CRF (bpm)	0.382	0.000 *	0.335	0.000 *
SBP (mmHg)	0.542	0.000 *	0.578	0.000 *
DBP (mmHg))	0.296	0.000 *	0.398	0.000 *

BMI = Body Mass Index; HDL-C = HDL-Cholesterol; LDL-C = LDL-Cholesterol; TG/HDL-C = Triglycerides to HDL-Cholesterol ratio; CRF = Resting Heart Rate; SBP = Systolic Blood Pressure; DBP = Diastolic Blood Pressure; MS: metabolic syndrome. * Significant: *p* < 0.05.

## Data Availability

Not applicable.
